# Central and peripheral blood pressures and arterial stiffness increase in hypoparathyroidism

**DOI:** 10.20945/2359-3997000000234

**Published:** 2020-03-30

**Authors:** Naim Pamuk, Tolga Akkan, Murat Dağdeviren, Arzu Or Koca, Esin Beyan, Derun Taner Ertuğrul, Mustafa Altay

**Affiliations:** 1 University of Health Sciences Turkey Keçiören Education and Research Hospital Department of Internal Medicine Ankara Turkey University of Health Sciences Turkey, Keçiören Education and Research Hospital, Department of Internal Medicine, Ankara, Turkey; 2 University of Health Sciences Turkey Keçiören Education and Research Hospital Department of Endocrinology and Metabolism Ankara Turkey University of Health Sciences Turkey, Keçiören Education and Research Hospital, Department of Endocrinology and Metabolism, Ankara, Turkey

**Keywords:** Arterial stiffness, blood pressure, hypoparathyroidism, hyperphosphatemia, pulse wave velocity

## Abstract

**Objective:**

The aim of the present study was to evaluate whether arterial stiffness is affected in the patients with hypoparathyroidism through pulse wave analysis (PWA).

**Subjects and methods:**

Sixty-three patients diagnosed with hypoparathyroidism and sixty volunteers were evaluated for the study. When 21 patients were excluded in the hypoparathyroidism group due to exclusion criteria, the research continued with 42 patients and 60 volunteers who are similar to the patients in terms of age, gender and body mass index (BMI). Fasting plasma glucose after 10 hours of fasting, creatinine, thyroid stimulating hormone (TSH), free thyroxine (fT4), albumin, calcium, phosphorus, magnesium, 25-OH vitamin D, parathormone (PTH) and urine calcium results in 24-hour urine for the patients in the hypoparathyroidism group were recorded. Evaluation of arterial stiffness was performed by Mobil-O-Graph 24h PWA device.

**Results:**

Systolic blood pressure (SBP) (p = 0.01), diastolic blood pressure (DBP) (p = 0.005), mean blood pressure (p = 0.009), central SBP (p = 0.004), central DBP (p = 0.01) and pulse wave velocity (PWV) (p = 0.02) were found higher in the hypoparathyroidism group. A positive correlation was detected between phosphorus level and SBP [(p = 0.03. r = 0.327)], central SBP [(p = 0.04, r = 0.324)] and PWV [(p = 0.003, r = 0.449)]. We detected that age and serum phosphorus levels were independent predictor variables for PWV (B = 0.014, p < 0.001 and B = 0.035, p < 0.001, respectively).

**Conclusion:**

We detected that hypoparathyroidism causes an increase in blood pressure and arterial stiffness. The most significant determinant factors were detected as advanced age and hyperphosphatemia. The patients diagnosed with hypoparathyroidism should be closely monitored and treatment planning should include to prevent the patients from hyperphosphatemia.

## INTRODUCTION

Hypoparathyroidism is an endocrine disorder characterized with lower levels or dysfunction of parathormone (PTH). Lower PTH levels within normal limits accompanied by hypocalcemia are classical for hypoparathyroidism. In addition, phosphorus levels may be higher. Hypoparathyroidism occurs due to insufficient hormone synthesis or accidental damage to or removal of the parathyroid glands during surgery or PTH receptor resistance very rarely. The most common cause of hypoparathyroidism in adults is surgical procedures on the neck and other causes include autoimmune diseases, genetic, infiltration, radiation and HIV infection (1).

Significant effects of the changes in PTH, calcium and phosphorus levels on cardiovascular system are well known. PTH receptors were shown in endothelial and myocardial cells and direct hypertrophic effects of PTH on myocardial cells were reported (2). Hypocalcemia may cause QT prolongation and life threatening arrhythmias (3). Although the mechanism is not certain, hypocalcemia is considered to reduce the contractility (4). Hyperphosphatemia is atypical laboratory finding of hypoparathyroidism and chronic kidney disease (CKD) (1,5). Persisting hyperphosphatemia is known to cause vascular calcification in in CKD (6,7). A previous study conducted on young adults with normal renal functions detected a significant association between phosphorus level and hypertrophy of the left ventricle (8). Furthermore, hyperphosphatemia may cause an increase in cardiovascular risk (9).

There are several invasive and non-invasive methods as well as scoring systems used to predict the increase in cardiovascular mortality and morbidity. Arterial stiffness is accepted as an independent risk indicator for cardiovascular diseases (10,11). Arterial stiffness may be detected through both invasive methods and non-invasive methods with proven reliability. Pulse wave velocity (PWV) is a practical and reliability -proven method which is measured non-invasively and demonstrates arterial stiffness indirectly (12,13).

There is limited number of studies focusing on effect of hypoparathyroidism on arterial stiffness parameters and conflicting results were obtained in such studies.

The aim of the present study was to evaluate whether arterial stiffness which is an independent parameter for cardiovascular diseases is affected in hypoparathyroidism through pulse wave analysis (PWA).

## SUBJECTS AND METHODS

### Study population and protocol

The present study enrolled the patients diagnosed with non-surgical or permanent surgical hypoparathyroidism (≥ 6 months) and healthy volunteers without any parathyroid gland disease or systemic disease referring to endocrinology and metabolism and internal medicine polyclinics of our hospital between January 2018 and December 2018.

Inclusion criteria were being adult (≥ 18 years), existence of non-surgical or permanent surgical hypoparathyroidism (≥ 6 months) diagnosis, having normal thyroid functions, corrected serum calcium level ≥ 7 mg/dL and asymptomatic presentation. Control group included healthy volunteers with normal levels of serum calcium, magnesium, phosphorus, parathormone levels and similar characteristics in terms of age, gender and body mass index (BMI).

**Figure 1:**
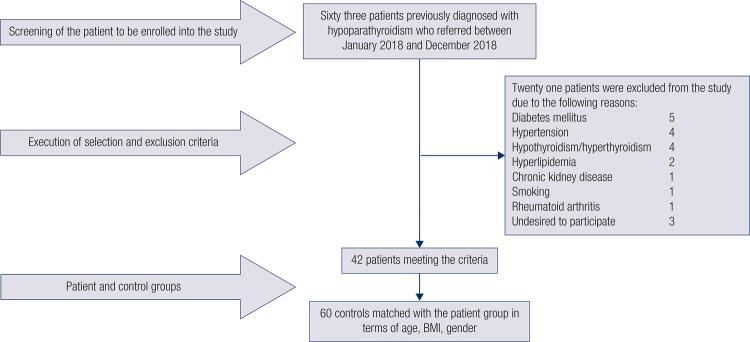
Determination of the patient group and the control group.

The exclusion criteria for both groups included the following; participants whose ages under 18, presence of any chronic disease (diabetes mellitus, hyperlipidemia, hypertension, coronary artery disease, peripheral artery disease, cerebrovascular disease, CKD, rheumatological disease, history of non-thyroid malignancy etc.), hypothyroidism or hyperthyroidism, smoking, pregnancy and lactation. In this context, 63 patients with hypoparathyroidism referring to our hospital were examined and 42 patients who accepted to participate into the study and 60 healthy volunteers were included into our study (Figure 1).

After history taking of 42 patients with hypoparathyroidism and 60 healthy volunteers, routine physical examinations were performed and findings were recorded. BMI was calculated through the formula weight (kg) / height (m^2^).

Laboratory tests of the patient and control groups including the following were recorded from computer data system of the hospital; fasting plasma glucose after at least10 hours of fasting (reference range 74-100 mg/dL), serum creatinine (0.57-1.11 mg/dL), thyroid stimulating hormone (TSH) (0.55-4 mIU/mL), free thyroxine (fT4) (0.78-1.48 ng/mL), albumin (3.5-5.2 g/dL), calcium (8.4-10.2 mg/dL), phosphorus (2.5-4.5 mg/dL), magnesium (1.8-2.4 mg/dL), 25-OH vitamin D (25-80 ng/mL), parathormone (15-65 pg/mL) and calcium in 24-hour urine. Corrected calcium was calculated in all participants [Corrected calcium = total calcium + 0.8 x (4-serum albumin)]. Corrected calcium value was multiplied with phosphorus and calcium-phosphate (CaXP) product levels were calculated.

Approval of the ethical committee was obtained for the research (2012-KAEK-15/1577). Verbal and written informed consent was obtained from all participants of the present study.

### Evaluation of pulse wave analysis and arterial stiffness

Arterial stiffness was detected through a Mobil-O-Graph PWA/ABPM device (I.E.M. GmBH, Stolberg, Germany) which performs cuff-based oscillometric method of measurement from brachial artery. Mobil-O-Graph PWA device was approved by blood pressure measurement BHS (British Hypertension Society) and ESH (European Society of Hypertension); and device reliability was demonstrated in comparison through invasive and non-invasive methods for PWA (14,15). The participants were allowed to rest in a silent room on sitting position for 15 minutes before arterial stiffness measurement. An adequate cuff was placed just above the elbow of the right arm. Four analyses were performed from the brachial artery on sitting position with a 5-minute interval. A bluetooth connection was established between PWA device and software recording the data from the device. Age, height and weight of the participant were entered into the software program. First, blood pressure was measured through a device and the pressure level which compresses the brachial artery completely was detected. A secondary complete compression was achieved on the brachial artery after a waiting period for 30 seconds. The point with no flow of the pulse waves which was sensed by specific sensors on the device was enlarged and scanned; and peripheral wave pulse data were created. The data created were classified as very high quality and low quality by the software; and very high-quality data were used in the present study. Central aortic pulse wave was calculated from pulse wave of the brachial artery through the software. PWA ended by calculation of the aortic pulse wave. Consequently, systolic blood pressure (SBP), diastolic blood pressure (DBP), mean arterial pressure (MAP), pulse, pulse pressure (PP), central SBP (cSBP), central DBP (cDBP), central PP (cPP), cardiac output (CO), peripheral vascular resistance, cardiac index (CI), body surface, augmentation pressure (AP), augmentation index adjusted according to 75 pulse/min (Aix@75), reflection magnitude and pulse wave velocity (PWV) data were obtained. These data were transferred into software program and recorded.

### Statistical analysis

Statistical analysis of the data was performed through SPSS 22.0 package program. Statistically significance level was accepted as p < 0.05. Descriptive statistical analyses of the patient and control groups were performed. Categorical data were reported as number and percentage. Kolmogorov-Smirnov test and histogram graphs were utilized to detect whether the data were compliant to normal distribution. Normally distributed data were expressed as mean and standard deviation. Non-normally distributed data were expressed in median and minimum-maximum. Student T test was used to evaluate normally distributed countable data whereas Mann-Whitney U test was used to evaluate non-normally distributed countable data. Chi-square test was implemented to compare categorical data. Correlation analyses were performed through Spearman’s and Pearson’s correlation tests. A multivariate regression analysis was done to detect independent variables predicting PWV. Since PWV data are not normally distributed, logarithmic transformation was performed.

## RESULTS

In the hypoparathyroidism group, 33 (78.6%) patients were followed due to surgical permanent hypoparathyroidism, 1 (2.4%) patient was followed because of autoimmune polyglandular syndrome, and 8 (19%) patients were followed because of primary idiopathic hypoparathyroidism.

There was not any statistically significant difference between two groups in terms of gender, age and BMI. Furthermore, we did not detect any statistically significant difference between the groups in terms of plasma glucose, serum creatinine, TSH, fT4, albumin, magnesium, 25-OH vitamin D levels (Table 1). Serum PTH, calcium and corrected serum calcium levels were significantly lower whereas phosphorus and CaXP product value were significantly higher in the hypoparathyroidism group when compared to the control group (Table 1). SBP, DBP, MAP, cSBP, cDBP and PWV were detected significantly higher in the hypoparathyroidism group (p = 0.01, p = 0.005, p = 0.009, p = 0.004, p = 0.01 and p = 0.02, respectively) (Table 2).

**Table 1:** Demographic and biochemical data in the hypoparathyroidism and control groups

	Hypoparathyroidism group (n = 42)	Control group (n = 60)	p
Age	40 (21-70)	42 (20-63)	NS
Female gender (%)	35 (83.3%)	49 (81.6%)	NS
BMI (kg/m^2^)	29.2 (19.4-37.1)	27.9 (18.4-38.6)	NS
FPG (mg/dL)	90 (74-98)	90 (75-99)	NS
Creatinine (mg/dL)	0.76 (0.57-1.01)	0.77 (0.6-1.03)	NS
fT4 (ng/dL)	1.19 (0.86-1.48)	1.12 (0.88-1.4)	NS
TSH (mU/L)	1.95 (0.62-4)	2.07 (0.66-3.98)	NS
Ca (mg/dL)	8.3 (7-10)	9.5 (8.9-10.2)	**<0.001**
Albumin (g/dL)	4.4 (3.9-5)	4.25 (3.7-5)	NS
cCa (mg/dL)	7.99 (7-9.22)	9.34 (8.64-10.04)	**<0.001**
Mg (mg/dL)	1.9 (1.6-3)	2 (1.9-2.4)	NS
P (mg/dL)	5.1 (3.1-6.9)	3.4 (2.5-4.5)	**<0.001**
CaxP (mg^2^/dL^2^)	40.3 (24.8-51.7)	31.7 (22.7-44.5)	**<0.001**
PTH (pg/mL)	3 (3-13.9)	37.9 (16-63.8)	**<0.001**
25-OH vitamin D (ng/mL)	23.5 (4.5-83.4)	21.64 (15.1-40.12)	NS
24-h urine Ca (mg/day)	150 (30-487)	-	-

NS: not significant; BMI: body mass index; FPG: fasting plasma glucose; fT4: free thyroxin; TSH: thyroid stimulating hormone; Ca: calcium; cCa: corrected calcium; Mg: magnesium; P: phosphorus; PTH: parathormone.

**Table 2:** Arterial stiffness data in the hypoparathyroidism group and the control group

	Hypoparathyroidism group (n = 42)	Control group (n = 60)	p
SBP (mmHg)	125.5 (103-146)	119.5 (103-139)	**0.01**
DBP (mmHg)	79.5 (67-96)	75 (53-89)	**0.005**
MAP (mmHg)	100 (85-117)	96 (78-119)	**0.009**
Pulse (pulse/min)	83 (63-117)	82 (54-108)	NS
PP (mmHg)	44.5 (23-67)	43.5 (26-69)	NS
Central SBP (mmHg)	117 (96-142)	110 (80-130)	**0.004**
Central DBP (mmHg)	80.5 (68-98)	77 (55-97)	**0.01**
Central PP (mmHg)	34.5 (19-56)	32.5 (20-56)	NS
CO (L/min)	5.1 (3.6-6.8)	4.8 (3.5-6.7)	NS
Peripheral resistance(s*mmHg/mL)	1.22 (0.9-1.73)	1.21 (0.81-1.56)	NS
Cardiac index (L/min/m^2^)	2.7 (1.9-4.3)	2.6 (1.9-3.7)	NS
AP (mmHg)	6 (2-24)	6 (2-20)	NS
Aix@75 (%)	29 (7-67)	26 (5-49)	NS
Reflection magnitude (%)	68 (53-79)	65.5 (34-80)	NS
PWV (m/s)	6.2 (4.7-10.2)	5.9 (4.3-8.5)	**0.02**

NS: not significant; SBP: systolic blood pressure; DBP: diastolic blood pressure; MAP: mean arterial pressure; PP: pulse pressure; CO: cardiac output; AP: augmentation pressure; Aix@75: augmentation index (corrected depending on 75 pulse/min); PWV: pulse wave velocity.

Correlation of arterial stiffness data with demographic data as well as biochemical parameters were reviewed in all participants. A positive significant correlation was detected between PWV and age (p < 0.001, r = 0.92), BMI (p < 0.001, r = 0.44) and FPG (p = 0.04, r = 0.925). There is not any significant correlation between two groups in terms of creatinine, TSH and fT4.

A significantly negative correlation was detected between serum calcium level and SBP, DBP, MAP, cSBP, cDBP, cPP and PWV. A significantly positive correlation was detected between serum phosphorus level and SBP, DBP, MAP, cSBP, cDBP, cPP and PWV. A significantly negative correlation was detected between serum parathormone level and SBP, DBP, MAP, cSBP, cDBP, cPP and PWV (Table 3).

**Table 3:** Correlation analysis results of arterial stiffness data of all participants

	Calcium (corrected)		Phosphorus		Ca x P		PTH	

	r	p	r	p	r	p	r	*p*
SBP (mmHg)	-0.29	0.003	.287	0.004	.249	0.01	-.241	0.015
DBP (mmHg)	-0.285	0.004	0.254	0.01	0.203	0.04	-0.196	0.048
MAP (mmHg)	-0.296	0.003	0.279	0.005	0.231	0.02	-0.204	0.04
PP (mmHg)	NS	NS	NS	NS	NS	NS	NS	NS
cSBP (mmHg)	-0.365	< 0.001	0.305	0.002	0.245	0.01	-0.283	0.004
cDBP (mmHg)	-0.253	0.01	0.239	0.02	NS	NS	NS	NS
cPP (mmHg)	-0.202	0.04	NS	NS	NS	NS	-0.203	0.04
AP (mmHg)	NS	NS	0.203	0.04	NS	NS	-0.203	0.04
Aix@75 (%)	NS	NS	NS	NS	NS	NS	NS	NS
PWV (m/s)	-0.243	0.01	0.269	0.006	0.256	0.009	NS	NS

NS: not significant; SBP: systolic blood pressure; DBP: diastolic blood pressure; MAP: mean arterial pressure; PP: pulse pressure; cSBP: central systolic blood pressure; cDBP: central diastolic blood pressure; cPP: central pulse pressure; AP: augmentation pressure; Aix@75: augmentation index (corrected depending on 75 pulse/min); PWV: pulse wave velocity; Ca: calcium; P: phosphorus; PTH: parathormone.

A positive correlation was detected between phosphorus level and SBP, cSBP and PWV (r = 0.327, p = 0.03; r = 0.324, p = 0.04 and r = 0.449, p = 0.003, respectively). A significantly negative correlation was detected between PTH and AP, cSBP and cAP (r = -0.328, p = 0.03; r = -0.341, p = 0.03 and r = -0.383, p = 0.01, respectively). The only parameter which has correlation with CaXP product was PWV (p = 0.006, r = 0.416). There was not any significant correlation detected between arterial stiffness parameters and serum calcium level as well as 24-hour urine calcium excretion.

We performed a linear regression analysis to detect independent variables to predict PWV. Since PWV does not distribute normally, we performed a logarithmic transformation for linear regression analysis. According to the univariate analysis result, we included age, BMI, serum low density lipoprotein cholesterol (LDL-C) and serum phosphorus levels to investigate whether such parameters are independent variables to predict PWV. We detected according to the multivariate linear regression analysis that age and serum phosphorus levels were independent predictor variables for PWV (B = 0.014, p < 0.001 and B = 0.035, p < 0.001, respectively) (Table 4).

**Table 4:** Multivariate linear regression analysis of independent predictor factors for PWV*

	B	95% CI	*p*
Age	0.014	0.013-0.015	<0.001
Serum phosphorus (mg/dL)	0.035	0.023-0.046	<0.001

* R^2^ = 0.89.

## DISCUSSION

We detected that PWV which indicates arterial stiffness as well as central and peripheral blood pressures were significantly higher in the patients with hypoparathyroidism. Furthermore, we also detected a positive correlation between PWV and serum phosphorus level, CaXP product; and age and serum phosphorus levels were the only independent variables which predict PWV.

Hypoparathyroidism presents with hypocalcemia and hyperphosphatemia similar to chronic kidney disease of which secondary hyperparathyroidism is detected (1,5). Hyperphosphatemia causes vascular calcification in the patients with CKD and thereby increases mortality of those patients (16). Sigrist and cols. evaluated vascular calcification through calcification score obtained from femoral arteries by multidetector computed tomography (CT) and performed pulse wave analysis through tonometric method in the cases with end stage kidney disease; they detected a positive correlation between femoral vascular calcification score and PWV as well as PP and a negative correlation between femoral vascular calcification score and DBP (17). Vascular calcification was found in association with CaXP product in the patients with CKD (18). Phosphorus uptake into the smooth muscle cells increases through “Na-P co-transporter (NPC)” along with the increase in extracellular phosphorus level. Increase intracellular phosphorus stimulates production of Core-binding factor alpha1(Cbfa-1) which is a transcriptor acting on osteogenic differentiation; and smooth cells gains osteoblast-like phenotypical characteristics. Consequently, hemodynamical effects appeared due to decrease of arterial elasticity include increase of systolic blood pressure, decrease in diastolic blood pressure and increase in pulse pressure (19,20). Beyond the information above, studies carried out demonstrated that hyperphosphatemia causes an increase in cardiovascular risk (9).

There are very limited number of studies focusing on hypocalcemia-hyperphosphatemia associated with hypoparathyroidism on cardiovascular system. Meena and cols. compared intima-media thicknesses of carotid artery, aorta and renal artery of 30 patients who were between 20 and 40 years of age and diagnosed with sporadic idiopathic hypoparathyroidism with healthy controls. Intima-media thicknesses of all three arteries were significantly higher in the hypoparathyroidism group when compared with the control group. However, there was not any correlation between intima-media thicknesses of the carotid artery, aorta and renal artery and calcium, phosphorus, CaXP product, vitamin D or PTH values. The most important limitations of their study were limited number of the cases and constricted age range of the patients (21). Agarwal and cols. obtained calcium score of the coronary artery through multidetector CT in their study conducted on the patient population with hypoparathyroidism. Calcification of the coronary artery with varying stages was detected in 3 patients in the hypoparathyroidism group whereas none of 40 volunteers in the control group had calcification of the coronary artery. Although the difference was not statistically significant, calcium score of the coronary artery of the hypoparathyroidism group was found higher and negatively correlated with serum calcium level (22). Such two studies conducted by using intima-media thicknesses of the carotid artery, aorta and renal artery as well as calcium score of the coronary artery support the increase of arterial stiffness detected in our study (23,24). However, a significantly positive correlation with phosphorus level detected in our study was not shown in those studies.

Literature screening revealed that there is not any study which compared PWV and blood pressure of the patients with surgical or non-surgical hypoparathyroidism with healthy population. The only study on arterial stiffness in the patients with hypoparathyroidism that we detected in the literature was conducted by Underbjerg and cols. who compared 56 patients diagnosed with non-surgical hypoparathyroidism and 30 patients diagnosed with pseudohypoparathyroidism in terms of BP and PWV. Calcium and PTH level were found higher in the pseudohypoparathyroidism group whereas there was not any difference between two groups in terms of CaXP product and phosphorus level. Although PWV was detected higher in the non-surgical hypoparathyroidism group, there was not any correlation between PWV and CaXP product, Ca and P levels. No difference of systolic, diastolic and mean AP was detected between two groups. Blood pressures were not correlated with CaXP product, PTH, Ca and P levels in the non-surgical hypoparathyroidism; however, a negative correlation was found between PTH level and diastolic blood pressure as well as mean arterial pressure in the pseudohypoparathyroidism group (25). There are similar and conflicting results of the aforementioned study when compared with the present study. Higher PWV in the non-surgical hypoparathyroidism group where mean PTH level is lower is in line with our study. However, lack of significant difference in terms of blood pressures when PWV is higher in the non-surgical hypoparathyroidism group and lack of correlation of PWV with any biochemical parameter conflict with our study. While one of the independent predictor factors of PWV is phosphorus, lack of difference in CaXP product and phosphorus detected by Underbjerg and cols. between two groups may be the cause for aforementioned conflict.

Since calcium levels usually decrease when phosphorus levels increase, we detected that blood pressures and PWV which is negatively correlated with calcium level are positively correlated with phosphorus level. Positive correlation of phosphorus level with SBP, cSBP and PWV in the hypoparathyroidism is completely in line with systolic blood pressure and arterial stiffness increase which are typical hemodynamical outcomes of vascular calcification developed because of hyperphosphatemia. Furthermore, such results obtained in the present study prove togetherness of BP increase and arterial stiffness increase as demonstrated in many studies (26-29).

The findings obtained in the present study indicate that hypoparathyroidism which is not identified as classical cardiovascular risk factor may be accepted as a cardiovascular risk factor when our findings are supported with further studies. Moreover, the positive correlation between PWV and phosphorus level and presence of hyperphosphatemia as an independent predictor for PWV reveal the necessity of protection of the patients with hypoparathyroidism from hyperphosphatemia as much as possible.

One of the major studies searching the effect of hypoparathyroidism on risk of cardiovascular disease and mortality was conducted by Underbjerg and cols. in 2018. The aforementioned study conducted in Denmark screened long term health records of 431 patients including 380 (88%) patients diagnosed with surgical hypoparathyroidism and 51 (12%) patients diagnosed with non-surgical hypoparathyroidism to investigate the association between risk of cardiovascular disease and mortality. A significantly positive association was found between mortality and higher phosphorus level and CaXP product whereas there was not any significant association found between hyperphosphatemia and risk of cardiovascular disease. It was shown that there is an association between increase of cardiovascular disease and deep hypocalcemia and frequency of hypercalcemia episodes (30). Inclusion of the patients with surgical hypoparathyroidism in aforementioned study is similar with the present study. Such study indicating the association between hyperphosphatemia and mortality as well as hypocalcemia and cardiovascular disease risk support our findings.

Vadiveloo and cols. compared 116 patients with surgical hypoparathyroidism and 106 patients with non-surgical hypoparathyroidism followed for 9 years with the control group in terms of cardiovascular disease risk and mortality. An increase was detected in cardiovascular disease risk and mortality in all hypoparathyroidism groups when compared with the control group. However, it was specified that such risk increase is caused by non-surgical hypoparathyroidism group and there was not any difference between surgical hypoparathyroidism group and the control group in terms of cardiovascular disease risk and mortality (31). It may be discussed that such study where no cardiovascular risk increase was detected in the surgical hypoparathyroidism group conflicts with the present study.

Almost a consensus exists on the hypothesis that cardiovascular disease risk and mortality increases in the patients diagnosed with non-surgical hypoparathyroidism. However, the articles in the literature report different results in the patients with surgical hypoparathyroidism in terms of cardiovascular disease risk and mortality. Our study included history of surgery in hypoparathyroidism group by 78.6%; and SBP, DBP, MAP, cSBP, cDBP and PWV were detected higher in the hypoparathyroidism group when compared with the control group. Results of the present study seems to support arterial stiffness and cardiovascular risk increase in the surgical hypoparathyroidism group. The positive correlation that we detected between phosphorus level and PWV may be considered that arterial stiffness increase may be caused by the increase in vascular calcification.

We preferred arterial stiffness measurement to detect cardiovascular risk in the patients with hypoparathyroidism. Since validity and reliability of this method was shown in many studies, we also used it to detect cardiovascular risk in some endocrinological diseases in our previous studies (32-35). Increase of the experiences and researches on this topic shows that arterial stiffness method which is easy and non-invasive to implement shall gradually enter into medical practice routine.

Our study has some limitations. Cross-sectional design is one of the limiting factors. Furthermore, we continued with the cases who had high blood pressure during arterial stiffness measurement even those patients were not diagnosed before, considering that possible etiology may be secondary hypertension due to hypoparathyroidism and cardiovascular effects. If such cases were the patients with primary hypertension who were not diagnosed before, they might have increased blood pressure and PWV average of the hypoparathyroidism group. Another limitation is lack of subgroup analysis due to limited number of the patients with non-surgical hypoparathyroidism. The strongest part of the present study was the finding that there was not any difference between two groups in terms of traditional cardiovascular risk factors such as age, gender, BMI, FPG, LDL-C and thyroid function tests.

Consequently, we detected an increase of arterial stiffness which is a reliable indicator for cardiovascular disease risk in hypoparathyroidism. We detected a positive correlation between hyperphosphatemia and PWV in the patients with hypoparathyroidism. Furthermore, we also detected that the independent predictor for PWV was phosphorus level. Therefore, the patients with hypoparathyroidism should be closely monitored for phosphorus-calcium balance and development of cardiovascular disease. We believe that our study would guide further comprehensive studies.
